# *Frankixalus*, a New Rhacophorid Genus of Tree Hole Breeding Frogs with Oophagous Tadpoles

**DOI:** 10.1371/journal.pone.0145727

**Published:** 2016-01-20

**Authors:** S. D. Biju, Gayani Senevirathne, Sonali Garg, Stephen Mahony, Rachunliu G. Kamei, Ashish Thomas, Yogesh Shouche, Christopher J. Raxworthy, Madhava Meegaskumbura, Ines Van Bocxlaer

**Affiliations:** 1 Systematics Lab, Department of Environmental Studies, University of Delhi, Delhi, 110 007, India; 2 Department of Molecular Biology & Biotechnology, Faculty of Science, University of Peradeniya, Peradeniya, Sri Lanka; 3 School of Biology and Environmental Science, University College Dublin, Belfield, Dublin, 4, Ireland; 4 Department of Life Sciences, The Natural History Museum, London, SW7 5BD, United Kingdom; 5 Department of Environmental Studies, Hindu College, University of Delhi, Delhi, 110 007, India; 6 Microbial Culture Collection, National Center for Cell Science, NCCS Complex, Pune University Campus, Ganeshkhind, Pune, 411 007, India; 7 Herpetology Department, American Museum of Natural History, Central Park West at 79th Street, New York, New York, 10024, United States of America; 8 Amphibian Evolution Lab, Biology Department, Vrije Universiteit Brussel (VUB), Pleinlaan 2, B-1050, Brussels, Belgium; Leibniz-Institute of Freshwater Ecology and Inland Fisheries, GERMANY

## Abstract

Despite renewed interest in the biogeography and evolutionary history of Old World tree frogs (Rhacophoridae), this family still includes enigmatic frogs with ambiguous phylogenetic placement. During fieldwork in four northeastern states of India, we discovered several populations of tree hole breeding frogs with oophagous tadpoles. We used molecular data, consisting of two nuclear and three mitochondrial gene fragments for all known rhacophorid genera, to investigate the phylogenetic position of these new frogs. Our analyses identify a previously overlooked, yet distinct evolutionary lineage of frogs that warrants recognition as a new genus and is here described as *Frankixalus* **gen. nov.** This genus, which contains the enigmatic ‘*Polypedates*’ *jerdonii* described by Günther in 1876, forms the sister group of a clade containing *Kurixalus*, *Pseudophilautus*, *Raorchestes*, *Mercurana* and *Beddomixalus*. The distinctiveness of this evolutionary lineage is also corroborated by the external morphology of adults and tadpoles, adult osteology, breeding ecology, and life history features.

## Introduction

The Old World tree frog family Rhacophoridae currently contains over 380 species in 17 genera [1,2], of which eight genera were recognized during the last decade [3–9]. Yet, despite the increasing interest in stabilizing anuran systematics (e.g., [5,6,10–12]), this large neobatrachian radiation still contains several nominal taxa with ambiguous generic placements that have not yet been investigated using molecular phylogenetic analyses. Four of the recently recognized rhacophorid genera—*Ghatixalus*, *Raorchestes*, *Beddomixalus* and *Mercurana* [5,7,9]—were described from the Western Ghats of India, a region which has witnessed a two-fold increase in the number of known tree frogs (from 21 to 43 species) over the past decade, supported by molecular evidence (e.g., [7,9,13,14]). Although rhacophorids of the Western Ghats have received considerable attention, the Northeast Indian members of this family have not yet been subjected to rigorous phylogenetic studies.

India is a country that contains high biodiversity, with the Western Ghats united in a single biodiversity hotspot with Sri Lanka, and the Northeast Indian region being part of two biodiversity hotspots, the Indo-Burma hotspot and the Himalaya hotspot [15,16]. The eight northeastern states, viz., Arunachal Pradesh, Assam, Manipur, Meghalaya, Mizoram, Nagaland, Sikkim and Tripura are particularly interesting, because they form the transitional zone between the Indian subcontinent and South-East Asia. Despite Northeast India being recognized as one of the major centers of amphibian diversity, so far only the caecilian fauna of this region has been studied within a phylogenetic framework [17,18].

During extensive fieldwork in four Northeast Indian states, we discovered several populations of tree hole breeding frogs. One of these species appeared to be the nominal taxon *Polypedates jerdonii*, originally described by Günther [19] from “Darjeeling”, West Bengal. Ever since its description, *Polypedates jerdonii* has been assigned to various widespread genera such as *Rhacophorus* [20–25], *Philautus* (*Kirtixalus*) [26] and *Philautus* (*Philautus*) [27]. This taxonomic uncertainty was presumably due to characters such as large snout-vent size and fairly extensive webbing between toes, which is comparable to members of the genera *Rhacophorus* and *Polypedates*. However, this species has been retained in the genus *Philautus* until today, because of “the large unpigmented eggs that strongly suggest that this species might have a direct development” [27].

To clarify the evolutionary position of the Northeast Indian tree hole breeding frogs, we performed phylogenetic analyses of nuclear and mitochondrial gene fragments totaling ~ 3800 characters for 86 taxa representing all major rhacophorid lineages. We demonstrate that these frogs form a distinct evolutionary lineage that warrants recognition as a new genus, and provide a detailed account of their breeding ecology, larval behavior, morphology, and osteology.

## Materials and Methods

### Ethics statement

This study was conducted with permissions and following guidelines from the responsible authorities in the State Forest Departments (Arunachal Pradesh CWL/G/13(17)/06-07/PT/3878-83 dated 05 June 2009; Manipur 3/22/2006-WL dated 03 March 2009; Meghalaya FWC.G/16 dated 19 February 2009, FWC.G/173 dated 23 March 2009 and Nagaland FOR/GEN-42/2006 dated 29 May 2007, FG-3/28/92 dated 06 April 2009), Ministry of Environment, Forest and Climate Change, Government of India. Our protocols of collection and research complied with the provisions of the Wildlife (Protection) Act 1972, Government of India. Specific methods of collection, euthanasia, tissue sampling and fixation used in our study followed the guidelines for use of live amphibians and reptiles in field research by the American Society of Ichthyologists and Herpetologists (ASIH) (http://www.asih.org/pubs/herpcoll.html; dated 13 March 2006), and were approved by the internal ethical committee of Department of Environmental Studies, University of Delhi.

### Field surveys and specimen collection

Field expeditions and sampling were carried out both during day and night in the monsoon months, from June to September of the years 2007–2010, in four Northeast Indian states: Arunachal Pradesh, Manipur, Meghalaya and Nagaland. Life history observations of ‘*Polypedates*’ *jerdonii* were done at night (between 18:00–22:00 hours) at Mawphlang Forest, Meghalaya (coordinates 25°26.29'N, 91°45.35'E, 1577 m asl) in June 2009. GPS coordinates were recorded using a Garmin 76CSx. Adult specimens were euthanized in 5% aqueous solution of tricaine methanesulfonate (MS222), fixed in 4% formalin and preserved in 70% ethanol. Immediately after euthanization, tissue samples were taken from the thigh muscle, preserved in 95% ethanol, and stored at –20°C in the Systematics Lab, Department of Environmental Studies, University of Delhi, India (SDBDU). Eggs and tadpoles (euthanized using MS222) were preserved in 3.5% formalin, buffered to pH 7.0 with sodium phosphate, monobasic (NaH_2_PO_4_H_2_O) and sodium phosphate, dibasic (anhydrous) (Na_2_HPO_4_). Voucher specimens are deposited in the Bombay Natural History Society Museum (BNHS), Mumbai, and Systematics Lab, University of Delhi (SDBDU).

### Taxon sampling and DNA protocols

This study includes 87 taxa, 86 rhacophorid taxa representing 73 species of all genera within the family, with a sampling emphasis on ‘*Polypedates*’ *jerdonii* (eight specimens) and an unknown but closely related species (three specimens). A dicroglossid served as the outgroup for phylogenetic analyses. For 62 taxa, sequence data were obtained from GenBank (S1 Table). For the other 25 specimens, genomic DNA was extracted from muscle tissue using a standard extraction protocol by Sambrook *et al*. [28], or the Qiagen DNeasy blood and tissue kit (Qiagen, Valencia, CA, USA) following the manufacturer’s protocol. Our total data matrix encompasses fragments of two nuclear genes (*RHOD*: ~ 300 bp and *RAG1*: ~ 550 bp) and three mitochondrial genes (~ 1400 bp: *12SrRNA*, *tRNA*^*VAL*^ and *16SrRNA*). Primer sets used for *RHOD* (Rhod1A, Rhod1D), *RAG1* (Rag1-C, Rag 1-E), *12SrRNA* and  *tRNA*^*VAL*^ (H3296, L2519), and *16SrRNA* (16Sar, 16Sbr) were published by Bossuyt and Milinkovitch [29], Biju and Bossuyt [30], Richards and Moore [31], and Simon *et al*. [32], respectively. Sequencing was performed on both strands using a BigDye Terminator v3.1 Cycle Sequencing kit and ABI 3730 automated DNA sequencer (Applied Biosystems). Newly generated sequences were checked and assembled in ChromasPro v1.34 (Technelysium Pty Ltd.), and submitted to GenBank under accession numbers KU169932–KU170018 and KU230453–KU230461 (S1 Table).

### Phylogenetic analyses

Sequences were aligned using ClustalX 1.64 [33]. Ambiguous sections were identified by eye for non-coding DNA and by comparison with amino acid sequences for coding DNA using MacClade v4.0 [34]. Phylogenetic estimations were obtained under Maximum Likelihood (ML) criteria and in a Bayesian framework. Maximum Likelihood analyses were performed with the concatenated dataset under the GTR+CAT model using RAxML 8.0 [35] on the Cipres Science Gateway [36]. The RAxML search was performed specifying 200 alternative runs on distinct random starting trees. Clade support was assessed by 1,000 rapid bootstrap replicates.

Bayesian analyses were performed with MrBayes 3.1.2 [37], using a GTR+G+I mixed model according to a gene-based data partition. Two parallel runs of four Markov chain Monte Carlo (MCMC) chains each were executed for 10 million generations, with a sampling interval of 500 generations and a burn-in corresponding to the first five million generations. Convergence of the parallel runs was confirmed by split frequency standard deviations (<0.01), and by potential scale reduction factors (~1.0) for all model parameters using Tracer v1.3 [38]. A Bayesian consensus phylogram and Bayesian Posterior Probabilities (BPP) were inferred from the last 10,000 sampled trees of both runs. Additional Bayesian analyses, using the above parameters, were done for the nuclear and mitochondrial datasets separately.

### Adult morphology

Determination of sex and maturity were done by examining the gonads through a small ventral incision. Measurements and associated terminology follow Biju *et al*. [39]; webbing formulae follow Savage and Heyer [40] as modified by Myers and Duellman [41]. The amount of webbing relative to subarticular tubercles is described by numbering the tubercles 1–3, starting from the toe discs. The term shank is used here to refer to the lower part of the leg containing the tibia, and thigh is used for the upper part containing the femur. Measurements of all specimens were taken by using a digital slide-caliper, or a binocular microscope with a micrometer ocular, to the nearest 0.1 mm. All measurements provided in the taxonomy section and S2 Table are in millimeters. See S1A File for abbreviations used in this section, and S2 File for details of specimens examined in the study.

### Adult Osteology

We used five osteological characters to compare the internal morphology of ‘*Polypedates*’ *jerdonii* with three relevant genera (*Gracixalus*, *Kurixalus* and *Philautus*) representing the evolutionary transitions in the backbone of the tree. These characters are: sphenethmoid; premaxillary teeth and structure of premaxillae; shape of the frontoparietals, vomerines, epicoracoidal bridge (= “base of the omosternum”, Liem [42]); sternum (= “metasternum”, Liem [42]); and carpal elements. The latter four characters were also used by Liem [42].

Osteological characterization was done for adults of ‘*Polypedates*’ *jerdonii*, *Philautus aurifasciatus*, *Gracixalus gracilipes* and *Kurixalus eiffingeri*. Two neutral buffered formalin preserved specimens of ‘*Polypedates*’ *jerdonii* (SDBDU 2009.1163 and SDBDU 2009.312a), and three 70% alcohol preserved specimens belonging to *Philautus aurifasciatus* (AMNH A24559), *Gracixalus gracilipes* (AMNH A163893) and *Kurixalus eiffingeri* (AMNH A14498) (the type species of the respective genera) were cleared and double stained following the procedure by Taylor and Van Dyke [43]. Initial dehydration was done in 100% ethanol, followed by submersion in alcian blue for cartilage staining. Excessive musculature was digested using an infusion of borax and trypsin, and the specimens were subsequently stained in alizarin red for bone visualization. Preparations were photographed and scored for bones and cartilage within 2–3 days after the staining procedure was completed. Osteological terminologies follow Trueb [44], Duellman and Trueb [45], Maglia *et al*., [46] Pugener and Maglia [47].

### Larval morphology

Morphological studies are based upon examination of four tadpoles. Two tadpoles BNHS 5975 and SDBDU 2009.1295 (stage 36) were examined with light microscopy for external morphology. Prior to morphological examination, preserved tadpoles were transferred via a series of 35%, 55% and 70% alcohol and kept in each of these for at least 24 hours [43]. Larvae were staged according to Gosner [48]. External larval morphological terminology and measurements were adapted from McDiarmid and Altig [49] and Bowatte and Meegaskumbura [50]. Measurements were rounded to the nearest 0.01 mm using a stereomicroscope with a micrometer ocular or a digital slide-caliper. Oral morphology and margins of papillae were visualized using blue ink. In two specimens (stage 36), incisions were made up to 2 mm deep on the sides of the mouth opening to observe the jaw sheaths. See S1B File for abbreviations used in this section. For museums and other frequently used terms, see abbreviations in S1C File.

### Nomenclatural acts

The electronic edition of this article conforms to the requirements of the amended International Code of Zoological Nomenclature (ICZN), and hence the new names contained herein are available under that Code from the electronic edition of this article. This published work and the nomenclatural acts it contains have been registered in ZooBank, the online registration system for the ICZN. The ZooBank LSIDs (Life Science Identifiers) can be resolved and the associated information viewed through any standard web browser by appending the LSID to the prefix "http://zoobank.org/". The LSID for this publication is: urn:lsid:zoobank.org:pub:2EBBC122-024A-4678-9B94-27AE2A873889. The electronic edition of this work was published in a journal with an ISSN, which has been archived and is available from the following digital repositories: PubMed Central, LOCKSS.

## Results and Discussion

### 1. Phylogenetic analyses reveal a distinct evolutionary lineage of tree hole breeding frogs

**Fig 1:**
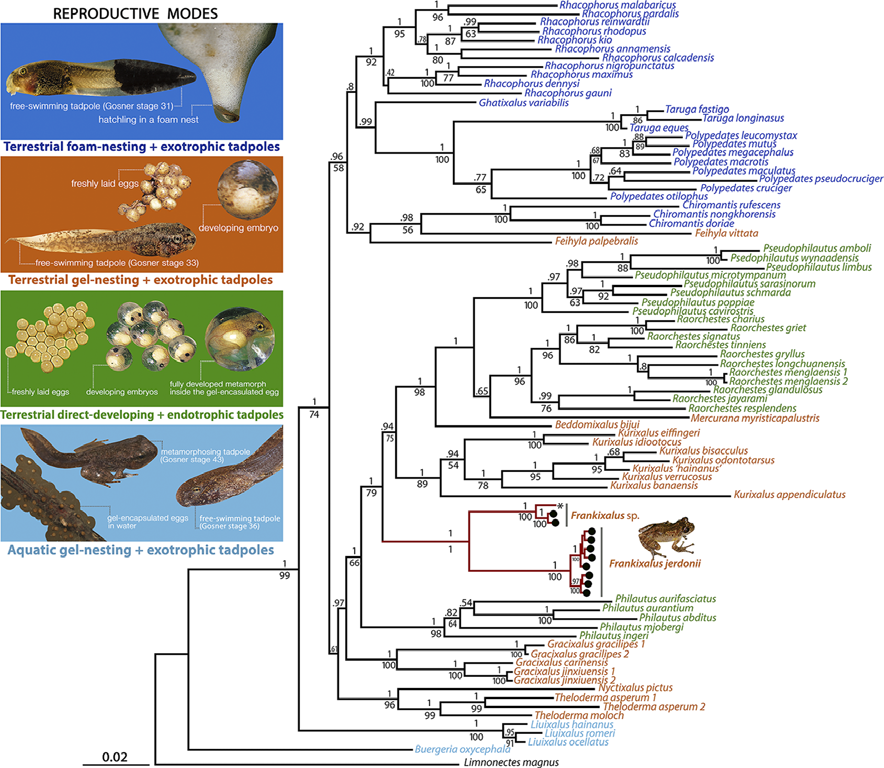
Bayesian consensus phylogram showing phylogenetic relationships among 86 taxa representing all known rhacophorid genera and one outgroup species. Numbers above the branches represent Bayesian Posterior Probabilities, numbers below the branches represent Maximum Likelihood bootstrap values. Clade representing *Frankixalus* gen. nov. is shown in red. The specimen that was assigned to “*Theloderma moloch*” by Li *et al*. [53] is indicated by an asterisk. Colors of taxa labels represent the reproductive modes: blue, terrestrial foam-nesting, exotrophic tadpoles; orange, terrestrial gel-nesting, exotrophic tadpoles; green, terrestrial direct-developing, endotrophic tadpoles; cyan, aquatic gel-nesting, exotrophic tadpoles. The new genus *Frankixalus* is also a terrestrial gel-nesting form.

Alignment of our DNA sequences resulted in a dataset of 3811 bp, 3174 of which could be aligned unambiguously. Maximum Likelihood analyses of the total dataset produced a single tree, which is very similar to the Bayesian consensus phylogram (Fig 1 and S1 Fig). The majority of the relationships obtained are in agreement with previous studies (e.g., [51,52]). In contrast with previous studies, however, *Theloderma moloch*, an adult from Namdapha, Arunachal Pradesh, which is close to the type locality (“Upper Renging”, Arunachal Pradesh), groups together with the other *Theloderma* species. The previously ambiguously assigned “*Theloderma moloch*” (larva voucher 6225Rao) from Li *et al*. [53] nested within the newly described genus (Fig 1).

Interestingly, our tree hole breeding frogs (represented by ‘*Polypedates*’ *jerdonii* and close relatives) form a distinct lineage that is the sister clade of a group containing the genera *Pseudophilautus*, *Raorchestes*, *Mercurana*, *Beddomixalus* and *Kurixalus*. Based on this phylogenetic evidence, we propose to allocate all frogs of this lineage to a new genus.

### Taxonomic treatment

Amphibia Linnaeus, 1758

Anura Fischer von Waldheim, 1813

Rhacophoridae Hoffman, 1932

Rhacophorinae Hoffman, 1932

***Frankixalus* gen. nov.** urn:lsid:zoobank.org:act:9DB458C3-9D5A-4058-842A-CEA7D41BA79F

(Figs 1–5)

**Fig 2:**
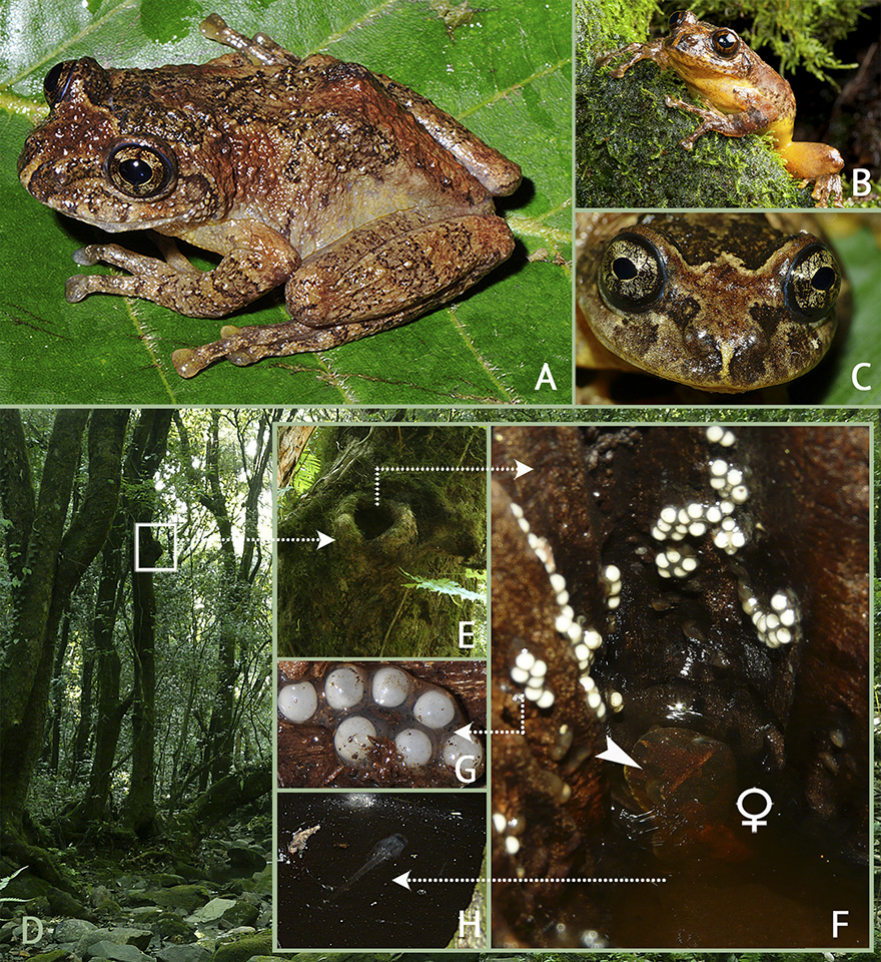
A–C, *Frankixalus jerdonii* in life. (A) dorsolateral view of an adult male (BNHS 5976), (B) an adult male (SDBDU 2009.271) emerging from a tree hole, (C) frontal view of an adult male (BNHS 5977). D–H, A composite showing the breeding habitat of *Frankixalus jerdonii*. (D) Evergreen forest at Mawphlang in East Khasi Hills district of Meghalaya state, (E) close-up of a tree hole opening located 3.4 meters above the ground, (F) oviposition site with eggs adhered to the inner vertical walls of the tree hole above the water level, and arrow pointing towards an adult female found submerged about 1 cm below the water surface, (G) unpigmented gel-encapsulated eggs, (H) premetamorphic larva inside the water-filled tree hole.

**Fig 3:**
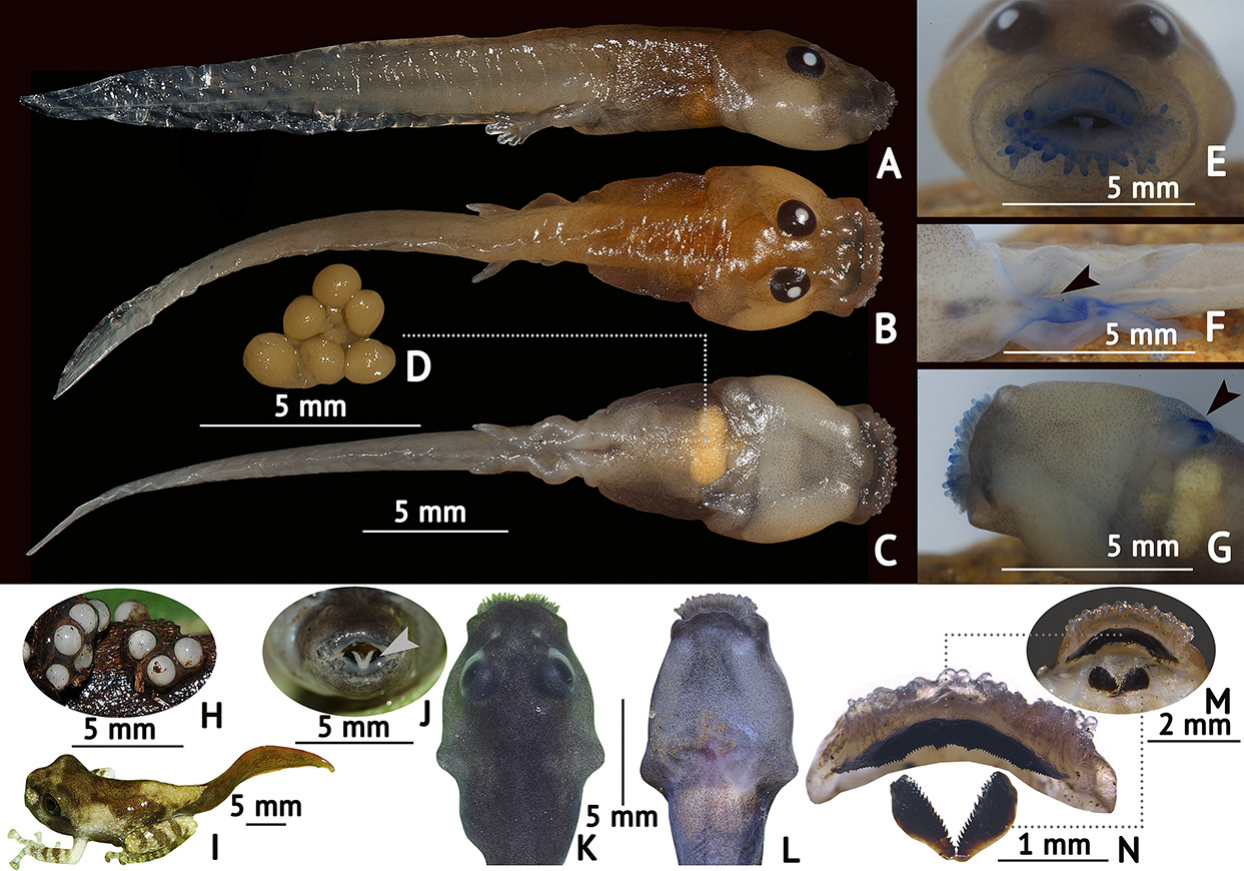
Various life history stages of *Frankixalus jerdonii*. (A) lateral, (B) dorsal, (C) ventral views of a preserved stage 36 tadpole, (D) unfertilised “nutritive” eggs found inside the dissected larval gut (mean diameter = 1.0 mm), (E) oral disc with papillae demarcating its margins, shown in frontal view of a stage 36 tadpole, (F) dextral vent tube, in ventral view of a stage 26 tadpole, (G) sinistral spiracular tube, in ventral view of a stage 36 tadpole, (H) gel-encapsulated eggs (mean diameter = 2.0 mm) found on the inside wall of a tree hole, (I) dorsolateral view of a stage 44 tadpole, (J) oral disc of a live stage 36 tadpole having a bifurcated muscular tongue, shown in frontal view, (K) dorsal, (L) ventral views of a live stage 35 tadpole, (M) serrated, inverted upper jaw of a stage 37 tadpole in ventral view, (N) serrated, V-shaped lower jaw of a stage 37 tadpole in ventral view.

**Fig 4:**
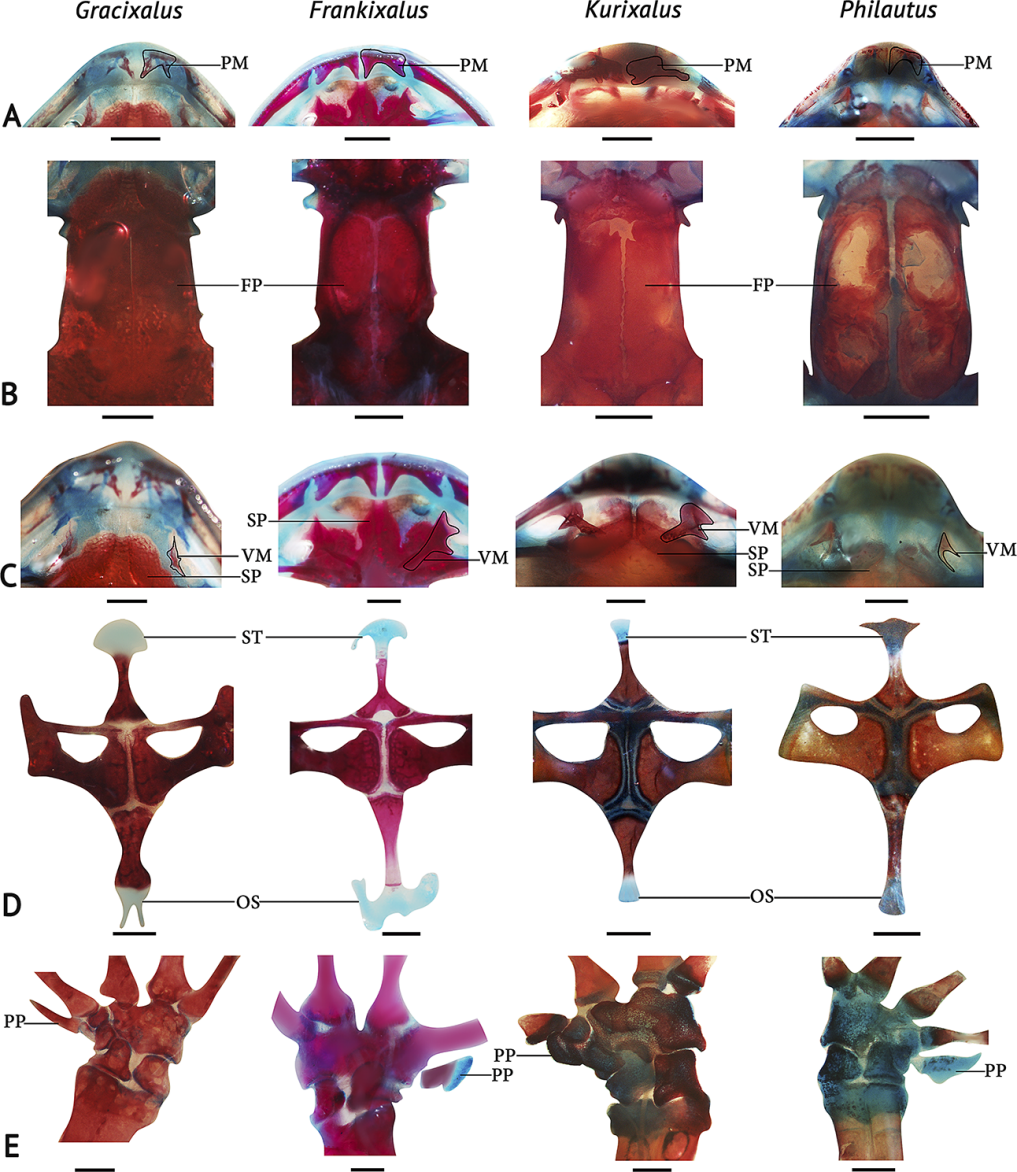
Comparison of osteological characters of four closely-related generic type species: *Gracixalus gracilipes* (AMNH A163893, an adult female, SVL 29.6 mm, from Vietnam), *Frankixalus jerdonii* (SDBDU 2009.1163, an adult male, SVL 41.6 mm, from Mawphlang), *Kurixalus eiffengeri* (AMNH A14498, an adult female, SVL 33.0 mm, from Taiwan) and *Philautus aurifasciatus* (AMNH A24559, an adult female, SVL 22.2 mm, from Indonesia). (A) shape of the premaxillae, (B) shape and development of frontoparietals, (C) structure of vomers, (D) ossified elements and shape of the pectoral girdles, (E) anatomy of the carpal elements. Abbreviations: FP, frontoparietal; OS, omosternum; PM, premaxilla; PP, prepollex; SP, sphenethmoid; ST, sternum; VM, vomer. All scale bars represent 2 mm.

**Fig 5:**
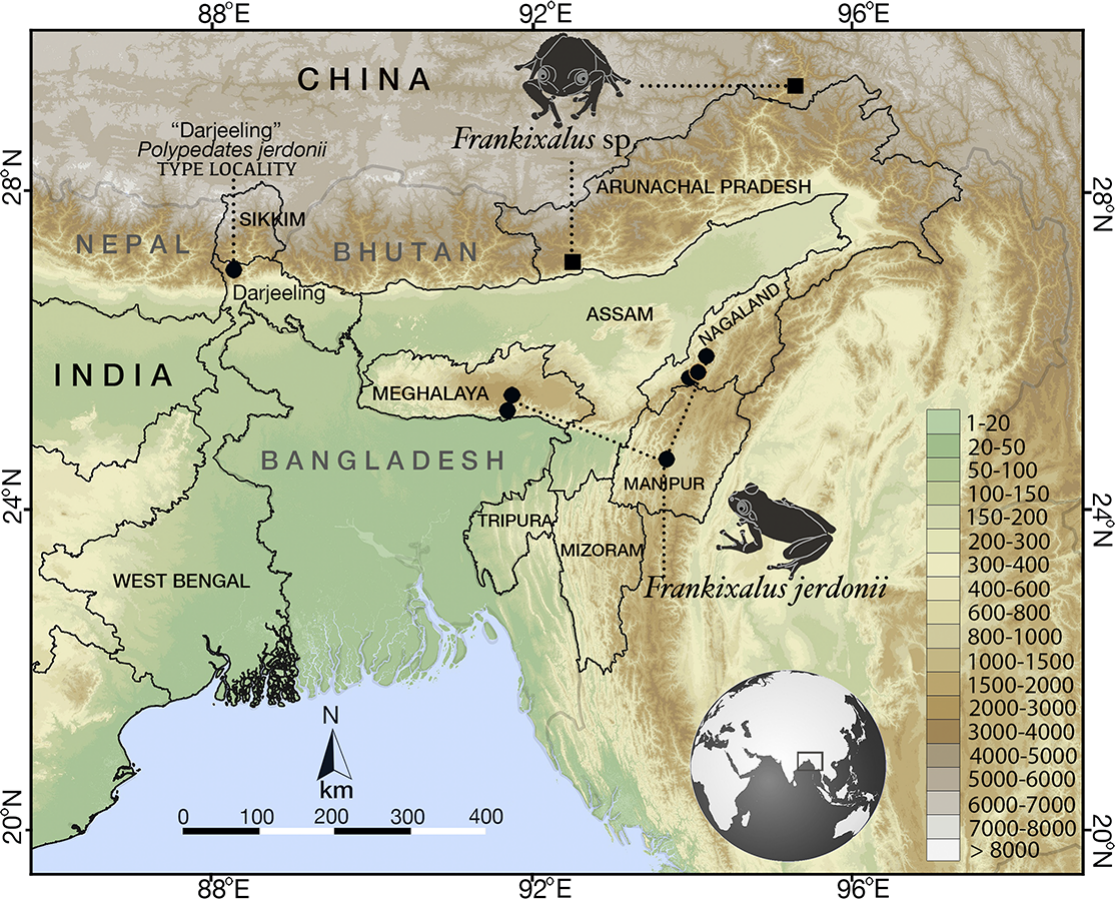
Geographic distribution of *Frankixalus* in Northeast India and China. Circle = *Frankixalus jerdonii*, square = *Frankixalus* sp.

#### Etymology

The genus is named after Prof. Franky Bossuyt of the Vrije Universiteit Brussel (Belgium), as a token of appreciation for his contribution to amphibian research and herpetology education, and in particular for the valuable role he played in the scientific career of SDB and IVB. The generic epithet is derived from the name ‘Franky’ (used as a noun in the nominative singular) in conjugation with the genus name ‘*Ixalus*’ Duméril & Bibron, 1841, often used as a suffix in rhacophorid generic names. For the purposes of nomenclature, the gender of this genus is male.

#### Suggested common name

Franky’s tree frogs

#### Type species

Polypedates jerdonii Günther, 1876

#### Diagnosis

We consider *Frankixalus* to consist of the most inclusive clade that contains *Frankixalus jerdonii* **comb. nov.** but not *Kurixalus eiffingeri*. *Frankixalus* currently contains two species, *F*. *jerdonii* (Günther, 1876) and a currently unidentified species.

*Frankixalus* can be distinguished from the other rhacophorid genera by the combination of the following characters: medium-sized adults (male SVL 37.1–42.1 mm, *N* = 11; female SVL 46.8 mm, *N* = 1), webbing medium (foot webbing: I2^–^–2^+^II1^+^–2^1^/_4_III1^+^–1^1^/_2_IV1^1^/_2_−1^+^V; hand webbing: I1–1^+^II1^+^–2^+^III2^–^–1^+^IV); creamy-white, gel-encapsulated eggs without pigmentation are laid in tree holes (phytotelm-breeding) where they also undergo development. The tadpole is oophagous and lacks keratinized tooth rows. The two currently included species are geographically restricted to high altitudes (approximately 1100–1600 m asl) in Northeast India and adjoining regions in China.

#### Comparison

*Frankixalus* differs from other rhacophorid genera as follows: *Breeding*, *oviposition and developmental strategies* (Fig 2): differs from *Philautus*, *Pseudophilautus* and *Raorchestes* by its free-living (exotrophic) tadpoles (vs. direct developing [endotrophic]); differs from *Chiromantis*, *Ghatixalus*, *Polypedates*, *Rhacophorus* and *Taruga* by its gel-encapsulated eggs (vs. eggs laid in foam nests); differs from *Beddomixalus*, *Buergeria*, *Feihyla*, *Liuixalus* and *Mercurana* by its phytotelm-breeding behavior, and oviposition on walls of tree holes (vs. terrestrial breeding and aquatic eggs in *Beddomixalus*, *Buergeria* and *Liuixalus*; leaf-breeding and oviposition on leaves in *Feihyla*; and terrestrial breeding and laid eggs mixed with mud in shallow pits in *Mercurana*). However, *Frankixalus* shares its breeding, oviposition and reproductive behavior with four other rhacophorid genera, namely *Gracixalus*, *Nyctixalus*, *Theloderma* and *Kurixalus*. *External morphology* (Fig 2): differs from all known rhacophorids by the combination of the following characters: outline of snout truncate in dorsal view, semi-circular in ventral view, vertical in lateral view (vs. not a combination of these characters), snout not protruding (vs. protruding). *Tadpole morphology* (Fig 3): differs from all known rhacophorids with exotrophic larvae by its absence of tooth rows (vs. present, except in *Rhacophorus vampyrus*) and presence of a sinistral, ventrally positioned spiracle (vs. sinistral, laterally positioned spiracle, except in *Rhacophorus vampyrus*). Presence of submarginal and marginal papillae shared with *Polypedates*, *Taruga*, *Beddomixalus*, *Mercurana*, *Gracixalus* and *Mercurana* (vs. absent in *Rhacophorus vampyrus*).

Adult members of the new genus *Frankixalus* are morphologically most similar to members of the genus *Kurixalus* due to comparable adult snout-vent size, SVL 37.1–42.1 mm, male (*N* = 11); SVL 46.8 mm, female (*N* = 1) (*Kurixalus* SVL 23.0–43.0 mm, male; 25.0–45 mm, female [54,55]); basal webbing on fingers, moderate webbing between toes. The breeding ecology of *Kurixalus* also resembles *Frankixalus*, especially the type species *Kurixalus eiffingeri*, which lays eggs on the inner walls of tree hollows or bamboo stumps with small water accumulations [56]. *Frankixalus* can be differentiated from *Kurixalus* by the following morphological characters: snout not protruding (vs. protruding); outline of snout rather truncate in dorsal view, semi-circular in ventral view (vs. pointed to rounded), dermal fringes absent (vs. dermal fringes present on forearm and tarsus [57–59] (S2 Fig). The differences between these genera are however most noticeable in osteology and tadpole morphology.

*Adult osteology* (Fig 4A–4E): Tri-radiate extension of the sphenethmoid develops surpassing the posterior margins of the nasal capsules in *Frankixalus* (Fig 4C). However, this is not apparent in *Gracixalus*, *Kurixalus* and *Philautus*, where the anterior margin of the sphenethmoid ends in a concave shape without extending beyond the nasal capsules. Premaxillary teeth are present in *Gracixalus*, *Philautus* and *Frankixalus*, but appear to be absent in *Kurixalus* (Fig 4A). Pars dentalis (of premaxillae) of *Kurixalus* is slightly medially depressed, but straight in the other three. *Frankixalus* has trapezoidal shaped frontoparietals, which are not fused medially, a condition also observed in *Gracixalus* and *Kurixalus*, but half oval-shaped frontoparietals were observed in *Philautus* (Fig 4B). The parieto-squamosal arch was absent in all four, which was an important feature highlighted by Liem [42] in distinguishing rhacophorid genera.

The structure of the dentary process (= “vomerine odontophore”) [42] of the vomers and presence/absence of vomerine teeth also showed differences and similarities between the four genera (Fig 4C). *Frankixalus* and *Kurixalus* have dentary processes extended towards the mid portion of the sphenethmoid; contrastingly, *Kurixalus* have vomerine teeth (2/3 blunt teeth on each); *Philautus* and *Gracixalus* lack a vomerine dentary process as well as vomerine teeth.

Considering the differences of omosternum and sternum (= “Metasternum”, Liem [42]), *Frankixalus* is the only genus among these genera to have a cartilaginous epicoracoidal bridge at the base of the omosternum, giving it a forked appearance (Fig 4D). *Philautus*, *Kurixalus* and *Gracixalus* possess omosternums with unforked, ossified bases. In all four genera, the bony stylus of the sternum extends posteriorly and ends in cartilaginous dilated distal ends. This is wider than its length in *Frankixalus* and has an irregular margin. Comparatively, *Kurixalus* and *Philautus* have smaller sized, cartilaginous distal ends, but this is fork-shaped in *Gracixalus* (Fig 4D).

Carpals (= “carpale/carpal elements”, Liem [42]) show slight differences among the four species (Fig 4E). Radiale and centrale, having a similar structure, are not different among the four. However, the prehallux shows considerable differentiation in developing into a distal prehallical element in *Gracixalus*, *Frankixalus* and *Philautus*, but not in *Kurixalus* (Fig 4E).

*Tadpole morphology* (Fig 3A–3M): Tadpoles of *Frankixalus* with relatively long bodies are moderately depressed in a lateral profile, which is similar to *Kurixalus* [60]. Bulbous medium-sized eyes are placed dorsally in *Frankixalus* and dorsolaterally in *Kurixalus*. In tree hole dwelling *Frankixalus*, a sinistral tubular spiracle is located ventrally and is clearly distinguishable as a protrusion from the ventral body wall with the spiracular opening located closer to the vent tube than the snout. However, in *Kurixalus*, a laterally placed sinistral spiracle is present (no tubular portion) with a rounded aperture [60].

Oral discs of *Frankixalus* and *Kurixalus* are elliptical and demarcated by rows of papillae. A major feature that differentiates these two genera is the existence of tooth rows. On *Frankixalus* the oral disc lacks tooth rows, but *Kurixalus* possesses three to five rows, depending on its developmental stage [60]. Keratinized lower and upper jaw sheaths are present in both *Frankixalus* and *Kurixalus*. In both, the upper jaw sheath (upper beak) is inverted U-shaped. However, the V-shaped lower jaw sheath is medially separated in *Frankixalus* (Fig 3M and 3N).

The tadpole of *Philautus* (= *Gracixalus*) cf. *carinensis* described by Wassersug *et al*. [61] appears to be most similar to *Frankixalus jerdonii* tadpoles by external morphology, but differs from *Frankixalus* by its denticle rows I/O and upper row closer to the margin of upper lip.

*Tadpole oophagy*: The presence of eggs in *Frankixalus jerdonii* larval intestines suggests that these tadpoles are oophagous in nature (see detailed notes under *Frankixalus jerdonii*). Three rhacophorid species where oophagous tadpoles have been reported so far are *Kurixalus eiffingeri* [56,60,62–63], *Philautus* (= *Gracixalus*) cf. *carinensis* [61] and *Rhacophorus vampyrus* [64,65].

### 2. Description of the name-bearing type

*Frankixalus jerdonii* (Günther, 1876) comb. nov.

#### Common name

Jerdon’s tree frog

#### Comments

Günther [19] described *Polypedates jerdonii* from “Darjeeling”, West Bengal, India on the basis of two syntypes [(NHM 1947.2.7.84 (ex BMNH 72.4.17.189), and NHM 1947.2.7.85 (ex BMNH 72.4.17.190)]. Since this species was first described, no additional specimens have been reported. Dubois [26] designated NHM 1947.2.7.84 (ex BMNH 1872.4.17.189) as the lectotype, without a formal description. We studied this specimen but found it to be poorly preserved (S3 Fig). Our extensive field surveys in the type locality “Darjeeling” and vicinities were unsuccessful in finding this species from the place of its original description. New collections of the nominal taxon in the present study are from East Khasi Hills district (Meghalaya state), Churachandarpur district (Manipur state) and Kohima district (Nagaland state). These specimens are morphologically similar to the type series.

A detailed description of the lectotype NHM 1947.2.7.84 (ex BMNH 1872.4.17.189) is provided in S2A File and also included in the morphometric measurement table (S2 Table).

#### Name-bearing type

Lectotype, NHM 1947.2.7.84 (ex BMNH 72.4.17.189), an adult female [26].

#### Type locality

“Darjeeling”, West Bengal, India.

#### Material examined

Measurements of thirteen specimens, collected from three states of Northeast India as part of this study and two museum specimens from “Darjeeling” (including the lectotype) are provided in S2 Table and S2 File.

#### Genetic divergence

Pairwise comparisons of the sequenced mtDNA 16S rRNA gene fragments of *Frankixalus jerdonii* and *Frankixalus* sp. show average uncorrected genetic divergence of 8.9% (8.4–9.5%, *N* = 16). Average uncorrected intraspecific genetic distance within *Frankixalus jerdonii* populations was 0.8% (0–1.7%, *N* = 8).

#### Color in life

BNHS 5976: Dorsally reddish-brown with a dark brownish-black X-shaped marking on the back (Fig 2A); BNHS 5977: Dorsum brownish-grey with a pair of brownish-black concave stripes that extend from behind the eyes to the vent, uniting in the middle and forming an X-shaped marking on the back; loreal and tympanic region dark brownish-grey with a red tinge; broad dark stripe on sides of the head; flanks light yellow; fore and hind limbs light brownish-grey with brownish-black cross-bands; iris reddish-brown, encircled with thin light blue outer ring; ventral surface yellowish-brown (Fig 2C); SDBDU 2009.1163: dorsum with yellowish-grey stripe between the eyes, a light reddish-brown triangular marking on the snout; iris golden brown, encircled with thin blue outer ring.

#### Secondary sexual characters

Male (NHM 1947.2.7.85): nuptial pad present on finger I, yellowish-white in color, vocal sac present, a pair of internal gular slits near the base of the lower jaw. Female (NMH 1947.2.7.84): a tube-like dermal extension of the cloaca present, eggs creamy-white, unpigmented (diameter 1.8 ± 0.8 mm, *N* = 10).

#### Intraspecific variation

Measurements representing morphological variations among eight specimens from different localities are provided in S2 Table.

#### Geographic distribution

*Frankixalus jerdonii* is widely distributed in three Northeast Indian states (Meghalaya, Manipur and Nagaland), and in the “Darjeeling” region of West Bengal. Meghalaya: East Khasi Hills district, Wahlynkien (Marai Kaphon), Cherrapunjee (25°16.673'N, 91°43.075'E, 1337 m asl), and Mawphlang forest (25°26.292'N, 91°45.348'E, 1577 m asl); Manipur: Churachandarpur district, Zaraengtung, Raenghzaeng village (24°38.790'N, 93°42.983'E, 1392 m asl); Nagaland: Kohima district, Sechüma village, Zubza (25°41.333'N, 94°01.767'E, 1470 m asl), Meriema village (25°43.0'N, 94°05.25'E, 1425 m asl), Seukwehii, Tseminyu village (25°55.541'N, 94°13.066'E, 1340 m asl); West Bengal: Darjeeling district, “Darjeeling” (27°03.617'N, 88°15.667'E, 1600 m asl) (Fig 5).

### 3. Natural history and breeding ecology

All males in our study were found on arboreal vegetation in montane evergreen forest (Mawphlang, Meghalaya), or secondary forests (Zaraengtung, Manipur and Zubza, Nagaland); males from Zubza were collected from inside bamboo poles with slits. Breeding activities of *Frankixalus jerdonii* take place between May–August. Males of *F*. *jerdonii* were heard calling at night (between 18:00–22:00 hours) from tree holes located at heights ranging from 0.8–5.5 m at Mawphlang forest, Meghalaya (in June 2009) soon after sporadic rain showers. The habitat at this locality is composed of an evergreen forest with sparse undergrowth, consisting of scattered shrubs and herbs (Fig 2D). The trunks of hardwood trees in these montane evergreen forests usually have large growths of bryophytes (Fig 2E). Amplexus was not observed. Freshly laid egg clutches (unpigmented, gel-encapsulated) were found adhering on the inner walls of a tree hole about 5 m above the ground (Fig 2F and 2G). In total, nine nest sites were observed in tree hollows, with tree diameters of about 10–30 cm (measured at the height of the hole). Occupied tree holes had openings oriented both horizontally (*N* = 5) and vertically (*N* = 4), usually with narrow openings, and contained water that ranged in depth from about 5–50 cm (volume of water contained ranging from 30–160 ml, *N* = 3). A deep layer of organic debris was observed at the bottom of some tree holes, and at two nest sites a dormant male was found submerged under water (Fig 2F). When disturbed, one male tightly wedged itself into a crevice in the bottom of the hollow. At another nest site, a female (not collected) was observed submerged in water. Eggs were observed between 0.3–10 cm above the water surface, were round, diameter measuring 2.0 ± 0.1 mm, *N* = 18, with a thick jelly layer of about 0.2–0.4 mm. Clutch size varied from 16–30 eggs per mass (2.5–5.6 cm, *N* = 7). During repeated surveys at the same site between 27–29 June 2009, we also observed tadpoles of various sizes (stages 10–44) inside the water-filled tree holes (Fig 2H).

### 4. Oophagy in tadpoles

At Mawphlang forest, we recorded 4–15 tadpoles inside each tree hole (*N* = 4), either resting motionless at the surface or at the bottom, but rapidly surfacing and diving occasionally to apparently gulp air. On being disturbed, they immediately hid under debris at the bottom of the cavity. Close morphological examination of tadpoles collected from the tree holes (stages 34–44) showed that they contained 3–19 eggs inside their intestines. Two individuals were further studied by making a small ventral incision: one (stage 36) contained at least 13 eggs (Fig 3D), and the other (stage 43) contained 19 eggs within their guts. Several of the eggs inside the abdomen were found intact (*N* = 10) and seem to have been eaten shortly before tadpole collection.

Oophagy in phytotelm-breeding frogs is an adaptation to live in nutrient deficient environments; our study shows that *Frankixalus jerdonii* tadpoles are indeed oophagous in nature. However, there is no direct evidence for the fact that these eggs are conspecific i.e. field observations of a female laying unfertilized eggs in the tree holes with tadpoles were not made. Nevertheless, suggestive evidence is available from (i) the presence of eggs in larval guts (Fig 3C), and (ii) females possessing an extension of the cloacal skin (like a tube) that might facilitate egg laying one at a time [60]. Future field studies are needed to gain additional knowledge on this matter.

### 5. Tadpole morphology

Descriptions of *Frankixalus jerdonii* tadpoles are based on stage 36 (*N* = 4) (Fig 3) and are compared with tadpoles of *Kurixalus*. *Frankixalus* tadpoles show similarities with other rhacophorid exotrophic larvae in several aspects (well-developed tail musculature, depressed snouts, elongated bodies, dextral vent tubes), except for a few significant characters in oral morphology (see the section ‘comparison‘).

The body is relatively long (BL 31.4% of TL) with clearly distinguishable wider anterior region and a narrower posterior region, ventrally flattened in a lateral profile. Bulbous medium-sized eyes (ED 20.7% of BW) are placed dorsally, located close to one another with a medial separation of 26.4% of BW. Nasolacrimal duct and elygium are absent. Snout appears depressed. Non-perforated, laterally placed narial depressions are present, with pigmentation visible around the pits. The narial depressions are located closer to the eyes than to the snout. Sinistral spiracular tube is ventrally positioned. The myotomic muscle masses, which are divided by V shaped septa, construct the larval tail region. The tail has unequal tail membranes, extending to the tip of the tail, which is rounded. The well-developed tail musculature is placed near the narrow posterior part of the body, and extends almost up to the tail tip. The tail fins arise at the distal end of the body, widen medially, and narrow again towards the tip. A dextral, well-developed vent tube with a medial opening is present.

The oral disc is elliptical and is demarcated by rows of submarginal and marginal papillae. The marginal papillae are continuous and possess both short and long blunt papillae, whereas the long ones are more concentrated along the lower labium. Lateral emarginations are present and the demarcation is apparent due to the presence of dense small papillae on the lateral sides of the oral disc. Blunt submarginal papillae are small in size, observed in 2–3 rows, and appear continuous throughout the lower labium. Tooth rows are absent, but keratinized lower and upper jaw sheaths represent the keratinized structures of the oral disc. Both upper and lower jaws have serrated margins on their posterior and anterior margins, respectively.

Similar to the case of *Kurixalus eiffingeri* [60], we assume that large amounts of yolk provide nourishment to the tadpoles even after hatching, until they develop mouthparts that facilitate oophagy.

Measurements of *Frankixalus jerdonii* tadpoles (stage 36, *N* = 4) are as follows: BH, 3.22; BL, 9.81; BW, 5.52; ED, 1.14; NN, 2.30; NP, 1.16; PP, 2.60; RN, 2.16; SS, 6.32; SU, 10.83; SVL, 10.40; TL, 31.28; VT, 17.98; TMH, 2.44; TMW, 2.21. Preserved specimens are deposited in SDBDU.

### 6. Conservation

The major threat for amphibians in Northeast India is disturbance of primary and secondary forests by ‘jhumming’ (slash and burn) with the purpose of cultivating crops. Several localities where *Frankixalus jerdonii* is reported to occur are highly disturbed and fragmented habitats. The population at Cheerapunjee in Meghalaya state was recorded from a secondary forest adjacent to a highly polluted Wahlynkien stream, individuals from Manipur were from tree stumps within a jhum field, and those from Nagaland were from a secondary forest. These threats are alarming, especially for species that have very specialized habitat requirements, such as availability of small water collections in tree holes that are crucial for their survival and reproductive success.

## Conclusion

Multiple lines of evidence from our study highlight the unique evolutionary position and life history features of *Frankixalus*. The description of this enigmatic lineage from the relatively unexplored northeast region of India not only emphasizes that part of this region’s biodiversity still remains poorly studied, but also underscores the need to replicate similar studies in other animal groups within this globally recognized biodiversity hotspot.

The description of this new rhacophorid genus adds to our knowledge on reproductive diversification in one of the most specious groups of neobatrachian amphibians. Such information is essential in understanding the evolution of reproductive strategies that allowed amphibians to occupy a broad variety of ecological niches (e.g., [11,30,52,66–69]).

## Supporting Information

S1 Fig Bayesian consensus phylogram of the total dataset showing phylogenetic relationships among 86 taxa representing all known rhacophorid genera and one outgroup species. Numbers above and below the branches represent Bayesian Posterior Probabilities obtained for the nuclear and mitochondrial datasets, respectively.
(PDF)

S2 Fig Snout shapes of *Frankixalus* and *Kurixalus eiffingeri*. A–C, *K*. *eiffingeri* (AMNH A14498, an adult female from Taiwan). (A) rounded in dorsal view, (B) rounded and protruding in ventral view, (C) rounded in lateral view; D–F, *Frankixalus jerdonii* (SDBDU 2009.1163). (D) truncate in dorsal view, (E) non-protruding semi-circular in ventral view, (F) vertical in lateral view.
(PDF)

S3 Fig Lectotype of *Polypedates jerdonii* (= *Frankixalus jerdonii*), NHM 1947.2.7.84 (ex BMNH 1872.4.17.189). (A) dorsal view, (B) ventral view, (C) lateral view of head, (D) ventral view of hand, (E) ventral view of foot, (F) schematic illustration of webbing on foot.
(PDF)

S1 File Abbreviations. (A) Material and methods adult morphology, (B) Material and methods larval morphology, (C) Museum and people.
(DOC)

S2 File Description of the lectotype of *Polypedates jerdonii* (= *Frankixalus jerdonii*), NMH 1947.2.7.84 (ex BMNH 1872.4.17.189), and additional specimens of *F*. *jerdonii* examined. (A) Description of lectotype (all measurements in mm), (B) Specimens of *Frankixalus jerdonii* examined.
(DOC)

S1 Table List of taxa and DNA sequences included in this study. (DOC)

S2 Table Morphometric measurements (in mm) of the specimens used in this study. Status of specimens is given after the Museum number: LT- Lectotype, PL-Paralectotype, RS- Referred specimens.
(DOC)
