# Supplementary material for: Frankixalus, a New Rhacophorid Genus of Tree Hole Breeding Frogs with Oophagous Tadpoles
Source: PLoS One. 2016 Jan 20;11(1):e0145727. doi: 10.1371/journal.pone.0145727 (PMC4720377; doi:10.1371/journal.pone.0145727)
Supplement: S1 Fig — Numbers above and below the branches represent Bayesian Posterior Probabilities obtained for the nuclear and mitochondrial datasets, respectively. (PDF) [file pone.0145727.s001.pdf]

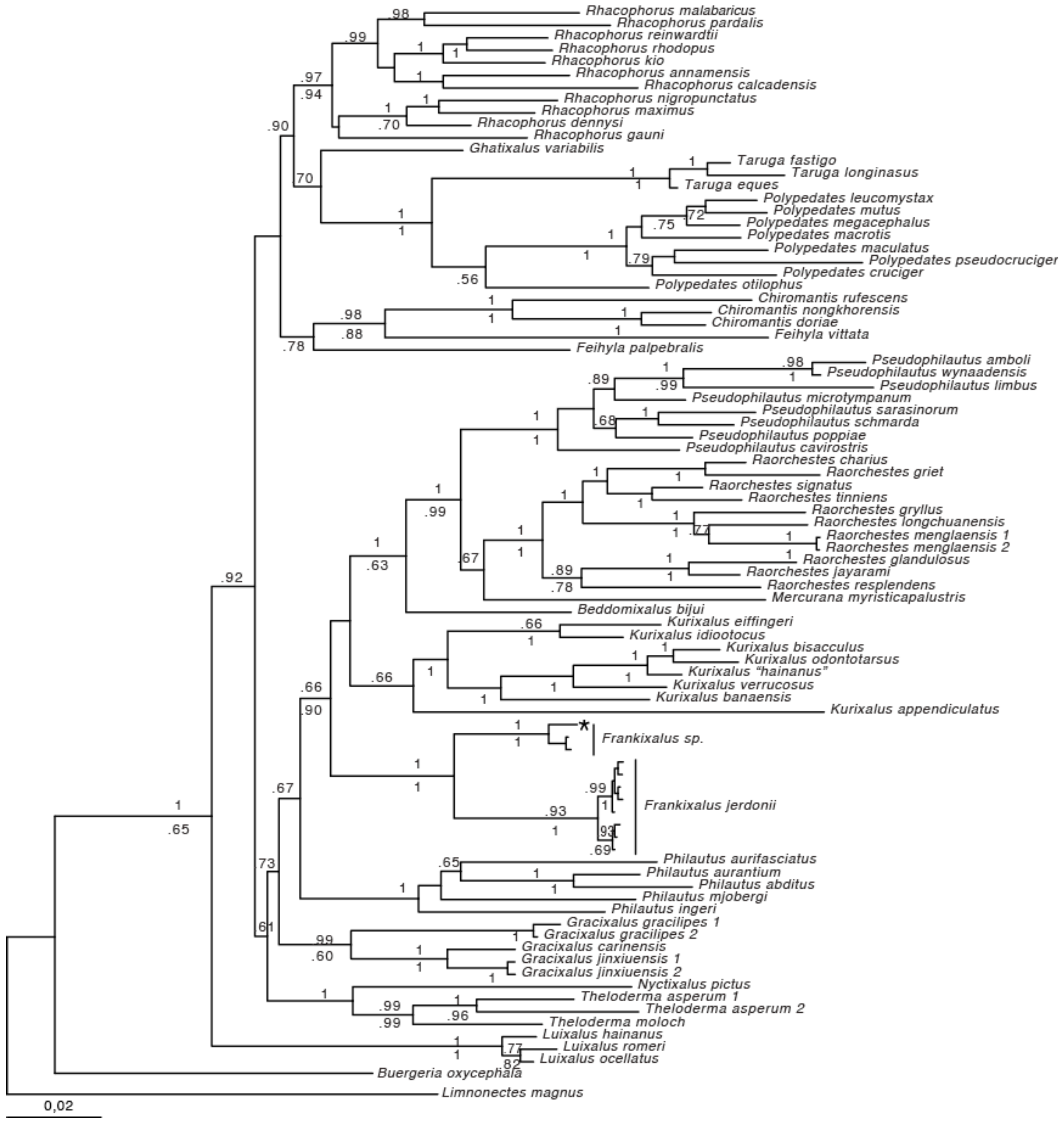

S1 Fig. Bayesian consensus phylogram of the total dataset showing phylogenetic relationships among 86 taxa representing all known rhacophorid genera and one outgroup species. Numbers above and below the branches represent Bayesian Posterior Probabilities obtained for the nuclear and mitochondrial datasets, respectively.
