# Supplementary material for: Frankixalus, a New Rhacophorid Genus of Tree Hole Breeding Frogs with Oophagous Tadpoles
Source: PLoS One. 2016 Jan 20;11(1):e0145727. doi: 10.1371/journal.pone.0145727 (PMC4720377; doi:10.1371/journal.pone.0145727)
Supplement: S2 Fig — A–C, K. eiffingeri (AMNH A14498, an adult female from Taiwan). (A) rounded in dorsal view, (B) rounded and protruding in ventral view, (C) rounded in lateral view; D–F, Frankixalus jerdonii (SDBDU 2009.1163). (D) truncate in dorsal view, (E) non-protruding semi-circular in ventral view, (F) vertical in lateral view. (PDF) [file pone.0145727.s002.pdf]

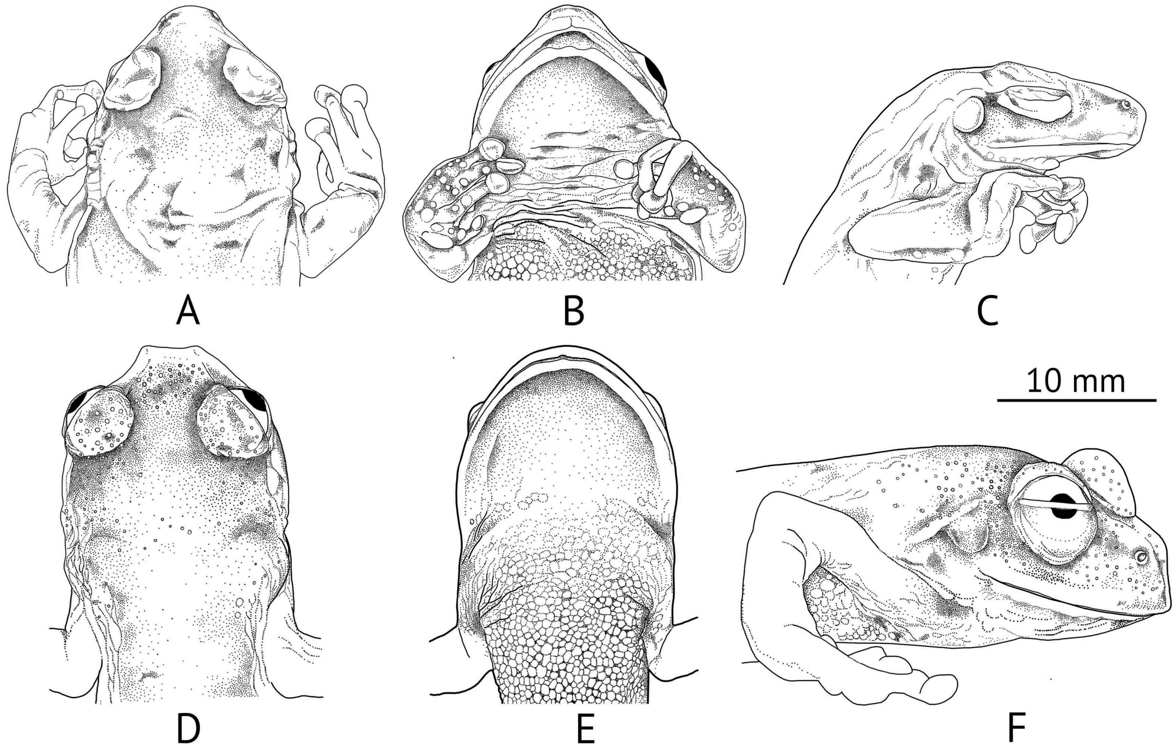

S2 Fig. Snout shapes of *Frankixalus* and *Kurixalus eiffingeri*. A–C, *K. eiffingeri* (AMNH A14498, an adult female from Taiwan). (A) rounded in dorsal view, (B) rounded and protruding in ventral view, (C) rounded in lateral view; D–F, *Frankixalus jerdonii* (SDBDU 2009.1163). (D) truncate in dorsal view, (E) non-protruding semi-circular in ventral view, (F) vertical in lateral view.
