# Supplementary material for: Frankixalus, a New Rhacophorid Genus of Tree Hole Breeding Frogs with Oophagous Tadpoles
Source: PLoS One. 2016 Jan 20;11(1):e0145727. doi: 10.1371/journal.pone.0145727 (PMC4720377; doi:10.1371/journal.pone.0145727)
Supplement: S3 Fig — (A) dorsal view, (B) ventral view, (C) lateral view of head, (D) ventral view of hand, (E) ventral view of foot, (F) schematic illustration of webbing on foot. (PDF) [file pone.0145727.s003.pdf]

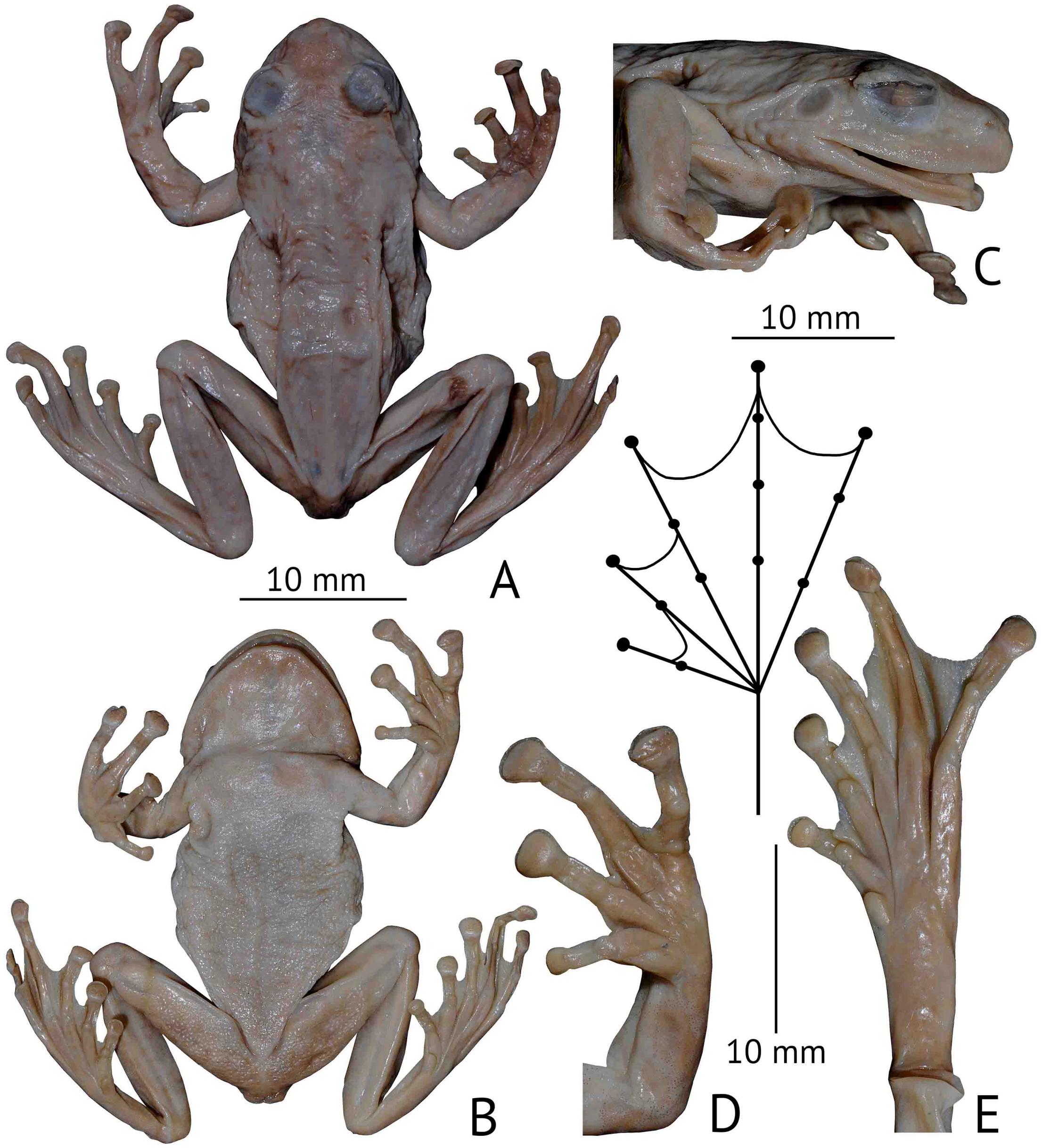

S3 Fig. Lectotype of *Polypedates jerdonii* (= *Frankixalus jerdonii*), NHM 1947.2.7.84 (ex BMNH 1872.4.17.189). (A) dorsal view, (B) ventral view, (C) lateral view of head, (D) ventral view of hand, (E) ventral view of foot, (F) schematic illustration of webbing on foot.
