# Supplementary material for: Frankixalus, a New Rhacophorid Genus of Tree Hole Breeding Frogs with Oophagous Tadpoles
Source: PLoS One. 2016 Jan 20;11(1):e0145727. doi: 10.1371/journal.pone.0145727 (PMC4720377; doi:10.1371/journal.pone.0145727)
Supplement: S1 File — (A) Material and methods adult morphology, (B) Material and methods larval morphology, (C) Museum and people. (DOC) [file pone.0145727.s004.doc]

**S1 File. Abbreviations.**

***A. Material and methods adult morphology:*** SVL (snout-vent length), HW (head width, at the angle of the jaws), HL (head length, from the rear of the mandible to the tip of the snout), MN (distance from the rear of the mandible to the nostril), MFE (distance from the rear of the mandible to the anterior orbital border), MBE (distance from the rear of the mandible to the posterior orbital border), SL (snout length, from the tip of the snout to the anterior orbital border), EL (eye length, horizontal distance between the bony orbital borders), IUE (inter upper eyelid width, shortest distance between the upper eyelids), UEW (maximum upper eyelid width), IFE (internal front of the eyes, shortest distance between the anterior orbital borders), IBE (internal back of the eyes, shortest distance between the posterior orbital borders), FAL (forearm length, from the flexed elbow to the base of the outer palmar tubercle), HAL (hand length, from the base of the outer palmar tubercle to the tip of the third finger), SHL (shank length), TL (thigh length), FOL (foot length, from the base of the inner metatarsal tubercle to the tip of the fourth toe), TFOL (distance from the heel to the tip of the fourth toe), FD (disc width of finger), FW (width of finger, measured at the base of the disc), TD (disc width of toe), TW (width of toe, measured at the base of the disc), digit number is represented by roman numerals I-V.

***B. Material and methods larval morphology:*** Maximum height of body (BH), maximum length of body (BL), maximum width of body (BW), maximum diameter of eye (ED), internarial distance (NN), naro-pupilar distance (NP), interpupilar distance (PP), rostro-narial distance (RN), distance from tip of snout to opening of spiracle (SS), distance from tip of snout to insertion of upper tail fin (SU), snout-vent length (SVL), total length (TL), distance from vent to tip of tail (VT), tail muscle height (TMH), and tail muscle width (TMW). Preserved specimens are deposited in the Systematics Lab, University of Delhi (SDBDU).

***C. Museum and people:*** For museums and frequently used terms, abbreviations are as follows: BNHS (Bombay Natural History Society, Bombay, India), DU (University of Delhi, Delhi, India), NHM (Natural History Museum, formerly British Museum (Natural History)), BMNH (British Museum (Natural History), London, United Kingdom), SDB (SD Biju), GS (Gayani Senevirathne), SG (Sonali Garg), SM (Stephen Mahony), Rachunliu G Kamei (RGK), Ashish Thomas (AT), YS (Yogesh Shouche), CJR (Christopher J Raxworthy), MM (Madhava Meegaskumbura), IVB (Ines Van Bocxlaer).
