# Supplementary material for: Frankixalus, a New Rhacophorid Genus of Tree Hole Breeding Frogs with Oophagous Tadpoles
Source: PLoS One. 2016 Jan 20;11(1):e0145727. doi: 10.1371/journal.pone.0145727 (PMC4720377; doi:10.1371/journal.pone.0145727)
Supplement: S2 File — (A) Description of lectotype (all measurements in mm), (B) Specimens of Frankixalus jerdonii examined. (DOC) [file pone.0145727.s005.doc]

**S2 File.** **Description of the lectotype of *Polypedates jerdonii* (= *Frankixalus jerdonii*), NMH 1947.2.7.84 (ex BMNH 1872.4.17.189), and additional specimens of *F. jerdonii* examined.**

***A. Description of lectotype (all measurements in mm):*** Adult female (SVL 46.8), body slender; head wider than long (HW 16.9, HL 15.7), its length **(**SL 6.7), longer than the horizontal diameter of the eye (EL 5.5); snout rather truncate in dorsal, ventrally semi-circular, vertical in profile, not protruding; canthus rostralis rounded, loreal region slightly concave, obtuse; interorbital distance (IUE 4.7), greater than width of the eye lids (UEW 4.3), and internarial distance (IN 4.4); nostrils dorsolaterally protruding, oval, equidistant from the eye (EN 3.3), and the snout tip (NS 3.3); tympanum fully exposed (TYD 1.6), circular; vomerine ridge distinct, obtuse, closer to choanae than each other; vomerine teeth absent; tongue deeply emarginated without median lingual process. Arms moderately long, thin, length (FAL 9.8) shorter then the hand (HAL 15.0); relative length of fingers I<II<IV<III; all finger tips dilated with wide oval discs (FDI 1.5, FWI 0.7; FDII 2.6, FWII 1.2; FDIII 2.9, FWIII1.6; WDIV 2.8, FWIV 1.6), circummarginal grooves present on all digit discs; all fingers with distinct lateral fringes on the inner and outer sides, webbing on fingers basal (I2^+^–2^+^II2^+^–3III3^–^–2^+^IV); subarticular tubercles prominently domed, circular, single, all present; thenar tubercle well developed, and inner and outer metacarpal tubercles absent; supernumerary tubercles weak on the base of finger I, II and II. Hind limbs relatively long, shank (SHL 20.6) longer than thigh (TL 19.7), and shorter than foot (FOL 22.0, TAFOL 31.1); toes moderately long and thick, relative lengths I<II<III=V<IV; tips of all toes with sub-circular discs, relative width of discs I<IV<II=V<III (TDI 1.6, TWI 0.9, TDII 2.3, TWII 0.9, TDIII 2.5, TWIII 1.0, TDIV 2.1, TWIV 0.8, TDV 2.3, TWV 0.9), all with circummarginal grooves; all toes with distinct lateral fringes on the inner and outer sides, toe webbing moderate (I2^–^–2^+^II1^+^–2^1^/_4_III1^+^–1^1^/_2_IV1^1^/_2_–1^+^V); subarticular tubercles all present, domed, and circular; inner metatarsal tubercle prominent, oval; tarsal glandular ridge, outer metatarsal, and supernumerary tubercles all absent.

Lectotype is clearly bleached of almost all pigmentation and skin texture (SI Figure 2), and cannot be reliably used to provide a comprehensive description. Hence, we are providing a description of this species based on the paralectotype NHM 1947.2.7.85 (ex BMNH 72.4.17.190).

*Skin of paralectotype NHM 1947.2.7.85 (ex BMNH 72.4.17.190)*. Skin on dorsal surface of head granular; lateral surfaces of head, flanks, dorsal surfaces of limbs appear weakly granular; dorsal surface of body, and ventral surfaces of the forelimbs, shanks and tarsus appear smooth; throat and chest covered with dense weak granules; abdomen and ventral and posterior surfaces of thighs covered with dense larger granules; lateral surfaces of the head posterior to the eye, and below the supratympanic fold, covered with enlarged glandular granules, except the tympanum which are smooth; supratympanic fold present, well developed, curving from the posterior border of the eye, over the tympanum to above the forelimb insertion; distinct nuptial pads present, surface microgranular, covering the dorsal surface of finger I extending to the base of the disc, and a smaller oval patch on the dorsal surface of the basal phalange of finger II.

*Coloration of paralectotype NHM 1947.2.7.85 (ex BNHS 72.4.17.190).* *In preservation:* A large brownish-purple blotch on the dorsum covering most of the head, and bifurcating just posterior to the level of the forelimbs insertions to form a broad rounded inverted V-shaped marking; the area between the darker inverted V-shaped marking is a slightly lighter shade of brownish-purple, as are the dorsal surface of the forearms, and thighs; dorsal surfaces of forearms, hands, shanks, tarsus and feet with faint to distinct transverse cross-bars; irregular brown mottling on the lower flanks; surfaces below the supratympanic folds and orbits, and lateral surfaces of the snout, including the canthus rostralis are dark brownish-purple; a light triangular marking on the dorsal surface of the snout, and two broad light lateral longitudinal bands extend from the rear of the orbits to the groin; ventral surfaces of the body and limbs primarily pale brownish-yellow, with extensive darker brown blotches covering the throat and chest.

***B. Specimens of Frankixalus jerdonii examined:*** West Bengal: Darjeeling district, “Darjeeling”, BMNH 1947.2.7.84 (ex 72.4.17.189), lectotype, an adult female, BMNH 1947.2.7.85 (ex 72.4.17.190), paralectotype, an adult male, collected by Jerdon. Meghalaya: East Khasi Hills district, Wahlynkien (Maria Kaphon), Cherrapunjee (25°16.673'N, 91°43.075'E, 1337 m asl), BNHS 5976, an adult male, collected on 28 May 2009 by SDB and RGK; Mawphlang Forest (25°26.292'N, 91°45.348'E, 1577 m asl), BNHS 5977, an adult male, collected on 1 June 2009 by SDB and RGK. Manipur: Churachandarpur district, Zarengtung, Raenghzaeng village (24°38.790'N, 93°42.983'E, 1392 m asl), SDBDU 2009.44–47 four adult males, collected on 13 May 2009 by RGK. Nagaland: Kohima district, Sechüma village, Zubza (25°41.333'N 94°01.767'E, 1470 m asl), SDBDU 2007.054–055, two adult males, collected on 14 June 2007 by RGK; Seukwehii, Tseminyu village (25°55.541'N 94°13.066'E, 1340 m asl), SDBDU 2009.362, an adult male, collected on 08 June 2009 by RGK; Meriema village (25°43.0'N 94°05.25'E, 1425 m asl), SDBDU 2007.060, collected on 14 June 2007 by RGK.
