# Supplementary material for: Frankixalus, a New Rhacophorid Genus of Tree Hole Breeding Frogs with Oophagous Tadpoles
Source: PLoS One. 2016 Jan 20;11(1):e0145727. doi: 10.1371/journal.pone.0145727 (PMC4720377; doi:10.1371/journal.pone.0145727)
Supplement: S1 Table — (DOC) [file pone.0145727.s006.doc]

**S1 Table.** **List of taxa and DNA sequences included in this study.**

| **Genus** | **Species** | **Collection Locality** | **Voucher No.** | **Acc. No.** |  |  |
| --- | --- | --- | --- | --- | --- | --- |
|  |  |  |  | *RHOD* | *RAG1* | *12SrRNA*, *tRNA^VAL^*, *16SrRNA* |
| **Rhacophoridae** |  |  |  |  |  |  |
| **Buergeriinae** |  |  |  |  |  |  |
| *Buergeria* | *oxycephala* | Hainan, China | SCUM 050267YJ | EU215556 | GQ285758 | EU215524 |
| **Rhacophorinae** |  |  |  |  |  |  |
| *Beddomixalus* | *bijui* | Kadalar, Kerala, India | SDBDU 2011.1006 | KU169946 | KU169970 | KU170017, KU169995 |
| *Chiromantis* | *doriae* | Laos | FMNH 255215 | GQ204657 | GQ204602 | GQ204772, GQ204721 |
| *Chiromantis* | *nongkhorensis* | Laos | FMNH 255378 | GQ204659 | GQ204604 | GQ204774, GQ204723 |
| *Chiromantis* | *rufescens* | Africa | CAS | GQ204660 | GQ204605 | GQ204775, GQ204724 |
| *Feihyla* | *palpebralis* | Lam Dong, Vietnam | KIZ 712 | GQ285792 | GQ285772 | GQ285681 |
| *Feihyla* | *vittata* | Yunnan, China | KIZ 0001 Rao | GQ285793 | GQ285774 | GQ285684 |
| *Frankixalus* | *jerdonii* | Raenghzaeng, Manipur, India | SDBDU 2009.46 | KU169947 | KU169971 | KU170018, KU169996 |
| *Frankixalus* | *jerdonii* | Cherrapunjee, Meghalaya, India | SDBDU 2009.271 | KU169948 | KU169972 | KU230453, KU169997 |
| *Frankixalus* | *jerdonii* | Mawphlang, Meghalaya, India | BNHS 5977 | KU169949 | KU169973 | KU230454, KU169998 |
| *Frankixalus* | *jerdonii* | Mawphlang, Meghalaya, India | SDBDU 2009.1166 | KU169950 | KU169974 | KU230455, KU169999 |
| *Frankixalus* | *jerdonii* | Tseminyu, Nagaland, India | SDBDU 2009.362 | KU169951 | KU169975 | KU230456, KU170000 |
| *Frankixalus* | *jerdonii* | Sechüma, Nagaland, India | SDBDU 2007.054 | KU169952 | KU169976 | KU230457, KU170001 |
| *Frankixalus* | *jerdonii* | Sechüma, Nagaland, India | SDBDU 2007.055 | KU169953 | KU169977 | KU230458, KU170002 |
| *Frankixalus* | *jerdonii* | Meriema, Nagaland, India | SDBDU 2007.060 | KU169954 | KU169978 | KU230459, KU170003 |
| *Frankixalus* | sp. | Sessa, Arunachal Pradesh, India | SDBDU 2009.1295 | KU169955 | KU169979 | KU230460, KU170004 |
| *Frankixalus* | sp. | Sessa, Arunachal Pradesh, India | SDBDU 2009.1261 | – | KU169980 | KU230461, KU170005 |
| *Frankixalus* | sp. | Motuo, Xizang, China | 6255Rao | GQ285782 | GQ285753 | GQ285679 |
| *Ghatixalus* | *variabilis* | Mukkurthi NP, Tamil Nadu, India | SDBDU 2008.4409 | KU169932 | KU169956 | KU170006, KU169981 |
| *Gracixalus* | *carinensis* | Sa Pa, Lao Cai, Vietnam | ROM39660 | GQ285788 | GQ285762 | GQ285670 |
| *Gracixalus* | *gracilipes* 1 | Pingbian, Yunnan, China | KIZ060821196 | EU924537 | EU924509 | EF564451, EF564523 |
| *Gracixalus* | *gracilipes* 2 | Mt. Dawei, Yunnan, China | 060821196RAO | GQ285789 | GQ285764 | GQ285668 |
| *Gracixalus* | *jinxiuensis* 1 | Mt. Dayao, Guangxi, China | KIZ 061210YP | EU215557 | GQ285763 | EU215525 |
| *Gracixalus* | *jinxiuensis* 2 | Jinxiu, Guangxi, China | KIZ060821013 | EU924543 | EU924515 | EF564452, EF564524 |
| *Kurixalus* | *appendiculatus* | Bukit Sarang, Sarawak, Malaysia | FMNH 267904 | – | JQ060911 | JQ060949, JQ060938 |
| *Kurixalus* | *banaensis* | Krong Pa, Gia Lai, Vietnam | ROM 32986 | GQ285781 | GQ285752 | GQ285667 |
| *Kurixalus* | *bisacculus* | Pua, Nan, Thailand | THNHM 10052 | – | JQ060897 | GU227280, GU227335 |
| *Kurixalus* | *eiffingeri* | Japan | A120 | AY880625 | – | DQ468673 |
| *Kurixalus* | ‘*hainanus*’ | Mt. Diaoluo, Hainan, China | HNNU A1180 | EU215578 | GQ285749 | EU215548 |
| *Kurixalus* | *idiootocus* | Lianhuachi, Taiwan, China | SCUM 061107L | EU215577 | GQ285751 | EU215547 |
| *Kurixalus* | *odontotarsus* | Mengyang, Jinghong, China | SCUM 060688L | EU215579 | GQ285750 | EU215549 |
| *Kurixalus* | *verrucosus* | Nagmung, Kachin, Myanmar | CAS 224381 | – | JQ060901 | GU227274, GU227329 |
| *Liuixalus* | *hainanus* | Mt. Diaoluo, Hainan, China | SCUM 060401L | GQ285785 | GQ285757 | GQ285671 |
| *Liuixalus* | *ocellatus* | Mt. Wuzhi, Hainan, China | HN0806045 | GQ285784 | GQ285755 | GQ285672 |
| *Liuixalus* | *romeri* | Mt. Shiwan, Guangxi, China | KIZ 061205YP | EU215559 | GQ285756 | EU215528 |
| *Mercurana* | *myristicapalustris* | Chathankod, Kerala, India | SDBDU 2011.849 | KU169945 | KU169969 | KU170016, KU169994 |
| *Nyctixalus* | *pictus* | Sarawak, Malaysia, Borneo | MVZ 239460 | GQ204666 | GQ204613 | GQ204783, GQ204732 |
| *Philautus* | *abditus* | Buon Luoi, An Khe, Vietnam | ROM33145 | GQ285794 | GQ285775 | GQ285673 |
| *Philautus* | *aurantium* | Mendolong, Malaysia, Borneo | FMNH233226 | GQ204642 | GQ204587 | GQ204756, GQ204705 |
| *Philautus* | *aurifasciatus* | Java, Indonesia | ZRC1.1.5267 | GQ204640 | GQ204584 | AY141805, GQ204702 |
| *Philautus* | *ingeri* | Sarawak, Malaysia, Borneo | FMNH239280 | AY880629 | GQ204588 | GQ204757, GQ204706 |
| *Philautus* | *mjobergi* | Sarawak, Malaysia, Borneo | FMNH252411 | GQ204644 | GQ204590 | GQ204759, GQ204708 |
| *Pseudophilautus* | *amboli* | Castle Rock, Maharashtra | SDBDU 2011.829 | KU169933 | KU169957 | KU170007, KU169982 |
| *Pseudophilautus* | *cavirostris* | Sri Lanka | WHT3299 | GQ204622 | GQ204561 | FJ788137, FJ788156 |
| *Pseudophilautus* | *limbus* | Haycock [Hiniduma], Sri Lanka | WHT2700 | – | GQ204553 | AY141779, GQ204668 |
| *Pseudophilautus* | *microtympanum* | Central Hills, Sri Lanka | WHT5065 | AF249126 | GQ204563 | AF249030, GQ204678 |
| *Pseudophilautus* | *poppiae* | Rakwana Hills, Sri Lanka | WHT2779 | GQ204616 | GQ204555 | FJ788136, FJ788155 |
| *Pseudophilautus* | *sarasinorum* | Sri Lanka | WHT2481 | GQ204614 | GQ204552 | AY141761, GQ204667 |
| *Pseudophilautus* | *schmarda* | Sri Lanka | WHT2715 | GQ204615 | GQ204554 | AY880617, GQ204669 |
| *Pseudophilautus* | *wynaadensis* | Kalpetta, Waynaad, Kerala, India | SDBDU 2010.334 | KU169934 | KU169958 | KU170008, KU169983 |
| *Polypedates* | *cruciger* | Sri Lanka | WHT2640 | GQ204632 | GQ204570 | GQ204746, GQ204687 |
| *Polypedates* | *leucomystax* | Java | ZRC 1.15269 | GQ204636 | GQ204574 | GQ204693 |
| *Polypedates* | *macrotis* | Malaysia | FMNH 239119 | GQ204638 | GQ204577 | GQ204748, GQ204695 |
| *Polypedates* | *maculatus* | Sri Lanka | WHKANT | GQ204637 | GQ204576 | GQ204747, GQ204694 |
| *Polypedates* | *megacephalus* | Mt. Daiyun, Fujian, China | SCUM 050508C | EU215582 | GQ285771 | EU215552 |
| *Polypedates* | *mutus* | Xishuangbanna, Yunnan, China | SCUM 37940C | EU215581 | GQ285770 | EU215551 |
| *Polypedates* | *pseudocruciger* | Chathankod, Kerala, India | SDBDU 2006.4770 | KU169935 | KU169959 | KU170009, KU169984 |
| *Polypedates* | *otilophus* | Malaysia | FMNH 239147 | GQ204639 | GQ204578 | GQ204749, GQ204696 |
| *Raorchestes* | *charius* | Coorg, Karnataka, India | SDBDU 2011.814 | KU169936 | KU169960 | KU170010, KU169985 |
| *Raorchestes* | *glandulosus* | Coorg, Karnataka, India | SDBDU 2011.817 | KU169938 | KU169962 | KU170012, KU169987 |
| *Raorchestes* | *griet* | Munnar, Kerala, India | SDBDU 2011.801 | KU169941 | KU169965 | KU169990 |
| *Raorchestes* | *gryllus* | Pac Ban, Tuyen Quang, Vietnam | ROM 30288 | GQ285796 | GQ285777 | GQ285674 |
| *Raorchestes* | *jayarami* | Munnar, Kerala, India | SDBDU 2011.807 | KU169940 | KU169964 | KU170013, KU169989 |
| *Raorchestes* | *longchuanensis* | Longchuan, Yunnan, China | 5RAO | GQ285795 | GQ285776 | GQ285675 |
| *Raorchestes* | *menglaensis* 1 | Lvchun, Yunnan, China | 060821286Rao | GQ285797 | GQ285778 | GQ285676 |
| *Raorchestes* | *menglaensis* 2 | Yunnan, China | KIZ060821286 | EU924544 | EU924516 | EU924626, EU924621 |
| *Raorchestes* | *resplendens* | Anamudi, Kerala, India | SDBDU 2009.1962 | KU169939 | KU169963 | KU169988 |
| *Raorchestes* | *signatus* | Avalanche, Tamil Nadu, India | SDBDU 2010.276 | KU169937 | KU169961 | KU170011, KU169986 |
| *Raorchestes* | *tinniens* | Munnar, Kerala, India | SDBDU 2010.274 | KU169942 | KU169966 | KU169991 |
| *Rhacophorus* | *anamensis* | Vietnam | FMNH 253934 | GQ204653 | GQ204598 | GQ204768, GQ204717 |
| *Rhacophorus* | *calcaneus* | Laos | FMNH 256465 | GQ204655 | GQ204600 | GQ204770, GQ204719 |
| *Rhacophorus* | *dennysi* | Jinxiu, Guangxi, China | KIZ060821050 | EU924548 | EU924520 | EF564467, EF564539 |
| *Rhacophorus* | *gauni* | Malaysia | FMNH 235047 | GQ204650 | GQ204596 | GQ204765, GQ204714 |
| *Rhacophorus* | *kio* | Xishuangbanna, Yunnan, China | SCUM 37941C | EU215562 | GQ285766 | EU215532 |
| *Rhacophorus* | *malabaricus* | Kalpetta, Wayanad, Kerala, India | SDBDU 2007.6019 | KU169943 | KU169967 | KU170014, KU169992 |
| *Rhacophorus* | *maximus* | Simao, Yunnan, China | KIZ060821140 | EU924552 | EU924524 | EF564476, EF564548 |
| *Rhacophorus* | *nigropunctatus* | Weining, Guizhou, China | SCUM 070657L | EU215563 | GQ285767 | EU215533 |
| *Rhacophorus* | *pardalis* | Malaysia | FMNH 231366 | GQ204647 | GQ204593 | GQ204762, GQ204711 |
| *Rhacophorus* | *reinwardtii* | Java | ZRC 1.1.5273 | GQ204656 | GQ204601 | GQ204771, GQ204720 |
| *Rhacophorus* | *rhodopus* | Mengyang, Jinghong, China | KIZ060821037 | EU215561 | EU924532 | EU215531 |
| *Taruga* | *eques* | Sri Lanka | WHT2741 | GQ204633 | GQ204571 | AY141801, GQ204689 |
| *Taruga* | *fastigo* | Sri Lanka | WHT2783 | GQ204634 | GQ204572 | AY141802, GQ204690 |
| *Taruga* | *longinasus* | Sri Lanka | WHTKAN1 | GQ204635 | GQ204573 | GQ204745, GQ204691 |
| *Theloderma* | *asperum* 1 | Jinping, Yunnan, China | KIZ060821201 | EU924562 | EU924534 | EF564449, EF564521 |
| *Theloderma* | *asperum* 2 | Malaysia | ZRC 1.1.9321 | GQ204661 | GQ204606 | GQ204776, GQ204725 |
| *Theloderma* | *moloch* | Arunachal Pradesh, India | SDBDU 2011.345 | KU169944 | KU169968 | KU170015, KU169993 |
| **Outgroup** |  |  |  |  |  |  |
| *Limnonectes* | *magnus* | Philippine Islands | 965 | DQ347373 | DQ347252 | DQ347314 |
