# Supplementary material for: Frankixalus, a New Rhacophorid Genus of Tree Hole Breeding Frogs with Oophagous Tadpoles
Source: PLoS One. 2016 Jan 20;11(1):e0145727. doi: 10.1371/journal.pone.0145727 (PMC4720377; doi:10.1371/journal.pone.0145727)
Supplement: S2 Table — Status of specimens is given after the Museum number: LT- Lectotype, PL-Paralectotype, RS- Referred specimens. (DOC) [file pone.0145727.s007.doc]

**S2 Table.** **Morphometric measurements (in mm) of the specimens used in this study.** Status of specimens is given after the museum number: LT- Lectotype, PL-Paralectotype, RS- Referred specimens.

|  | **Sex** | **Locality** | **Museum Number** | **SVL** | **HW** | **HL** | **TYD** | **SL** | **IUE** | **UEW** | **EL** | **FAL** | **HAL** | **FDIII** | **FWIII** | **TL** | **SHL** | **FOL** | **TAFOL** | **TDIV** | **TWIV** |  |
| --- | --- | --- | --- | --- | --- | --- | --- | --- | --- | --- | --- | --- | --- | --- | --- | --- | --- | --- | --- | --- | --- | --- |
| *Frankixalus jerdonii* | | |  |  |  |  |  |  |  |  |  |  |  |  |  |  |  |  |  |  |  |  |
|  | M | “Darjeeling” (PLT) | NHM 1947.2.7.85 | 42.1 | 15.2 | 13.5 | 1.8 | 5.6 | 4.4 | 3.7 | 5.2 | 8.8 | 14.4 | 2.5 | 1.3 | 18.2 | 19.5 | 20.7 | 29.6 | 2.4 | 1.5 |  |
|  | M | Mawphlang (RS) | BNHS 5977 | 40.6 | 14.9 | 13.9 | 1.5 | 5.8 | 4.0 | 3.5 | 4.9 | 7.9 | 12.8 | 2.3 | 1.1 | 17.5 | 18.4 | 18.8 | 29.1 | 2.5 | 1.2 |  |
|  | M | Cherrapunjee (RS) | BNHS 5976 | 37.1 | 13.5 | 12.5 | 1.3 | 5.8 | 4.2 | 3.1 | 4.5 | 7.1 | 11.9 | 2.3 | 1.0 | 16.8 | 17.9 | 18.1 | 26.3 | 2.2 | 1.0 |  |
|  | M | Raenghzaeng (RS) | SDBDU 2009.44 | 40.0 | 14.1 | 13.4 | 1.9 | 5.3 | 4.0 | 3.5 | 4.7 | 8.2 | 13.2 | 2.6 | 1.1 | 17.5 | 18.2 | 18.4 | 28.5 | 2.1 | 0.9 |  |
|  | M | Raenghzaeng (RS) | SDBDU 2009.45 | 41.5 | 14.5 | 13.6 | 1.9 | 5.9 | 4.0 | 3.6 | 4.9 | 8.7 | 13.8 | 2.2 | 1.0 | 18.1 | 18.9 | 19.3 | 28.9 | 2.1 | 0.8 |  |
|  | M | Raenghzaeng (RS) | SDBDU 2009.47 | 37.9 | 13.9 | 12.5 | 1.7 | 5.6 | 4.0 | 3.5 | 4.4 | 7.2 | 12.6 | 2.3 | 1.1 | 16.5 | 17.8 | 18.2 | 26.9 | 2.1 | 1.0 |  |
|  | M | Raenghzaeng (RS) | SDBDU 2009.46 | 41.5 | 14.4 | 13.9 | 1.9 | 5.8 | 4.1 | 3.8 | 5.1 | 8.0 | 13.8 | 2.6 | 0.9 | 17.8 | 18.2 | 19.8 | 29.7 | 2.1 | 0.9 |  |
|  | M | Sechüma (RS) | SDBDU 2007.054 | 37.8 | 13.2 | 12.5 | 1.7 | 5.2 | 4.1 | 3.2 | 4.4 | 7.4 | 12.9 | 2.2 | 1.1 | 16.9 | 17.8 | 18.1 | 26.4 | 2.2 | 1.2 |  |
|  | M | Sechüma (RS) | SDBDU 2007.055 | 37.7 | 13.3 | 12.4 | 1.7 | 5.3 | 4.0 | 3.3 | 4.2 | 7.1 | 12.6 | 2.5 | 1.2 | 16.3 | 17.8 | 18.3 | 25.6 | 2.1 | 1.1 |  |
|  | M | Tseminyu (RS) | SDBDU 2009.362 | 41.1 | 14.2 | 13.7 | 1.8 | 5.8 | 4.1 | 3.1 | 5.0 | 8.4 | 13.1 | 2.8 | 1.3 | 16.5 | 17.8 | 19.8 | 29.4 | 2.3 | 1.0 |  |
|  | M | Meriema (RS) | SDBDU 2007.060 | 41.4 | 14.3 | 13.8 | 1.9 | 5.6 | 4.2 | 3.1 | 5.3 | 8.5 | 13.2 | 2.8 | 1.1 | 16.1 | 18.1 | 19.3 | 29.5 | 1.5 | 0.7 |  |
|  |  |  | **Average** | **39.9** | **14.1** | **13.2** | **1.7** | **5.6** | **4.1** | **3.4** | **4.8** | **7.9** | **13.1** | **2.5** | **1.1** | **17.1** | **18.2** | **19.0** | **28.2** | **2.1** | **1.0** |  |
|  |  | **Standard deviation** | | **1.9** | **0.6** | **0.6** | **0.2** | **0.2** | **0.1** | **0.3** | **0.4** | **0.6** | **0.7** | **0.2** | **0.1** | **0.7** | **0.5** | **0.9** | **1.5** | **0.3** | **0.3** |  |
|  | F | “Darjeeling” (LT) | NMH 1947.2.7.84 | 46.8 | 16.9 | 15.7 | 1.6 | 6.7 | 4.7 | 4.3 | 5.5 | 9.8 | 15.0 | 2.9 | 1.6 | 19.7 | 20.6 | 22.0 | 31.1 | 2.1 | 1.5 |  |
